# Adequate Pelvic Lymph Node Dissection in Radical Cystectomy in the Era of Neoadjuvant Chemotherapy: A Meta-Analysis and Systematic Review

**DOI:** 10.3390/cancers15164040

**Published:** 2023-08-10

**Authors:** Krystian Kaczmarek, Bartosz Małkiewicz, Artur Lemiński

**Affiliations:** 1 Department of Urology and Urological Oncology, Pomeranian Medical University, Powstańców, Wielkopolskich 72, 70-111 Szczecin, Poland; 2 University Center of Excellence in Urology, Department of Minimally Invasive and Robotic Urology, Wroclaw Medical University, 50-556 Wroclaw, Poland

**Keywords:** bladder cancer, neoadjuvant chemotherapy, radical cystectomy, lymphadenectomy, survival analysis

## Abstract

**Simple Summary:**

Bladder cancer is the 10th most common cancer in the world, and its incidence is gradually rising worldwide. Currently, radical cystectomy with pelvic lymphadenectomy and preoperative chemotherapy is the mainstay of treatment for muscle-invasive bladder cancer. The therapeutic role of lymphadenectomy and neoadjuvant chemotherapy is similar in terms of treatment of micrometastatic disease. Therefore, we performed a systematic review and meta-analysis on the impact of adequate lymphadenectomy on patient survival in the era of the multidisciplinary approach. Our results showed a lack of association between adequate lymphadenectomy and overall survival in patients exposed to neoadjuvant chemotherapy.

**Abstract:**

Radical cystectomy (RC) with pelvic lymphadenectomy (PLND) serves as the gold-standard treatment for muscle-invasive bladder cancer (MIBC). Numerous studies have shown that the number of lymph nodes (LN) removed during RC could affect patient prognosis. However, these studies confirmed the association between PLND and survival outcomes prior to the widespread adoption of neoadjuvant chemotherapy (NAC). Consequently, this study aimed to investigate the prognostic role of PLND in patients previously pretreated with NAC. A systematic review and meta-analysis were performed using PubMed, Web of Knowledge, and Scopus databases. The selected studies contained a total of 17,421 participants. The meta-analysis indicated a significant correlation between adequate PLND and overall survival in the non-NAC group. However, a survival benefit was not observed in patients undergoing RC with preoperative systemic therapy, regardless of the LN cut-off thresholds. The pooled HR for ≥10 and ≥15 LN were 0.87 (95% CI 0.75–1.01) and 0.87 (95% CI 0.76–1.00), respectively. The study results suggest that NAC mitigates the therapeutic significance of PLND, as patients pre-treated with NAC no longer gain oncological benefits from more extensive lymphadenectomy. This highlights the analogous roles of NAC and PLND in eradication of micrometastases and in prevention of distal recurrence post-RC.

## 1. Introduction

Primary surgical treatment of muscle-invasive bladder cancer (MIBC) involving radical cystectomy (RC) and pelvic lymph node dissection (PLND) results in recovery for 50% of patients. PLND has long been a standard part of MIBC management. In 1950, Leadbetter and Cooper were the first to recommend regional lymph node dissection at the time of cystectomy. They observed a high recurrence and metastasis rate in patients who underwent cystectomy without PLND, leading to a high mortality rate. The unsatisfactory treatment outcomes were attributed to the unrecognized spread of the bladder cancer (BC) into pelvic lymphatics [1]. These assumptions from Leadbetter’s report were confirmed by Kerr and Colby’s study, which presented a series of patients with BC who underwent cystectomy, ureterosigmoidostomy, and bilateral PLND. They found lymph node metastasis in 40% of the patients, despite the absence of palpable lymph nodes during surgery. Moreover, they emphasized that intraoperative and postoperative morbidity was acceptable [2]. However, less optimistic results concerning lymph node metastasis were reported in an autopsy study by Wallmeroth et al. They found that 92% of patients with MIBC had metastases to the prevesical or pelvic lymph nodes, and only 12% had nodal dissemination as the sole metastatic manifestation [3]. Currently, despite the aggressive early approach towards MIBC, approximately 25% of patients show pathologically confirmed lymph node metastases at the time of cystectomy [4,5]. However, even in the presence of lymph node metastases, long-term overall survival (OS) is achievable and has been observed. Approximately 60% of patients survive five years after RC. The longer OS in patients undergoing RC with PLND is attributed to the two primary aspects of nodal dissection: staging instrument and therapy. Better staging enables the identification of node-positive patients who may benefit from adjuvant chemotherapy, whereas the therapeutic role is linked to the removal of micrometastatic disease in clinically node-negative patients [6,7]. However, the procedure of PLND remains underutilized, and various anatomical boundaries of PLND along with thresholds for the number of lymph nodes as surrogate markers of adequate oncological dissection have been proposed [8]. Additionally, the contemporary standard treatment for patients with MIBC now includes neoadjuvant chemotherapy (NAC). Preoperative systemic treatment should be offered to all pT2-4bN0M0 patients eligible for cisplatin-based regimens, providing a similar therapeutic benefit in eliminating micrometastatic disease as PLND [9]. Therefore, it is currently unclear if the therapeutic benefit of PLND is preserved in patients undergoing NAC. In response, this study aimed to perform the first meta-analysis to clarify the potential oncological benefit of PLND in the NAC era.

## 2. Materials and Methods

### 2.1. Search Strategy

Two authors (K.K. and A.L.) independently conducted an electronic bibliographic search of the Web of Knowledge, Scopus, and PubMed databases. The following search keywords were utilized: (overall survival OR survival OR prognosis) AND (radical cystectomy) AND (neoadjuvant chemotherapy) AND (pelvic lymph node dissection OR lymph node counts OR lymphadenectomy). Moreover, the references in the included studies, along with those of a previous systematic review, were examined [9,10,11,12]. All the studies included in this analysis were published in English. The searches were conducted without a time restriction, with the last search run on 20 June 2023.

### 2.2. Inclusion Criteria 

The Preferred Reporting Items for Systematic Reviews and Meta-analysis guidelines were followed, and the Population, Intervention, Comparator, Outcome, and Study design approach was used to describe study eligibility [13,14]. Studies were deemed relevant to this meta-analysis if they compared patients diagnosed with MIBC who underwent RC alone or were preoperatively treated with NAC and had “adequate” PLND to those who had “inadequate” PLND at the time of RC. The objective was to determine the role of “adequate” PLND as a predictor of OS, using multivariable Cox proportional hazards regression analysis. Meeting abstracts, case reports, editorials, commentaries, letters, and review articles were excluded. 

Studies were selected based on the following criteria:

- Large studies that included more than 100 patients;
- Studies that included patients with cT2-4aN0M0 BC;
- Studies that utilized at least one of two criteria of “adequate” PLND at the time of RC: ≥10 and/or ≥15 DLNs;
- Studies that assessed prognostic value of adequate PLND in patients who underwent RC alone and in those who preoperatively received NAC;
- Studies that provided hazard ratios (HRs) from multivariable Cox proportional hazards regression analysis with their corresponding 95% confidence intervals (CIs) or studies that provided enough data to calculate Cls;
- Studies that excluded patients who underwent concurrent nephroureterectomy due to a concomitant upper tract urinary carcinoma.

### 2.3. Systematic Review Process

**Figure 1:**
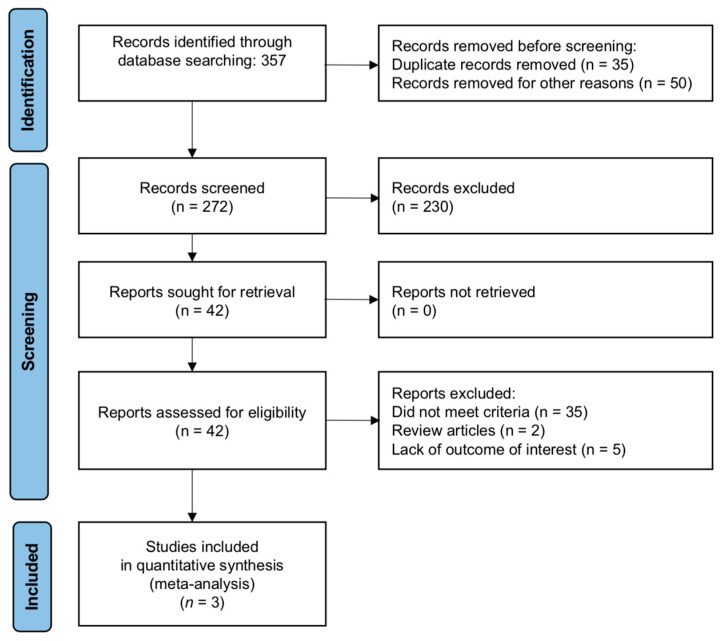
Flow chart of study selection process.

Following the removal of duplicates, the titles and abstracts of 272 studies were screened by two authors (K.K. and A.L.) for initial inclusion. Full-text evaluations were conducted on 42 potentially relevant studies, three of which met all the inclusion criteria and were included in the meta-analysis. The studies incorporated into the analysis were published between 2018 and 2022. Any disagreements between the two reviewers were resolved through discussion. All authors agreed on the final list of included articles. A PRISMA flow diagram of the study selection process is presented in Figure 1.

### 2.4. Quality of Data Assessment

All included articles were observational series. The quality of the studies was evaluated using the Newcastle–Ottawa Scale and involved rating each study using the star system, which comprises three aspects: selection of the study groups, comparability of the groups, and evaluation of the outcome of interest. The quality scores for all the included studies ranged from 7 to 8 stars (Table 1). Trials scoring ≥7 stars were deemed of sufficient quality for meta-analysis. 

### 2.5. Outcome Measures

Pooled HRs were used to assess the prognostic role of adequate PLND at the time of RC. HRs for OS were calculated and compared between patients with and without adequate PLND. The outcomes of individual studies were evaluated based on exposure to NAC.

### 2.6. Statistical Analysis

The *I*^2^ statistic was employed to detect heterogeneity across the included studies. The *I*^2^ statistic describes the percentage of variability in effect estimates, with a value >50% considered indicative of heterogeneity. In the absence of heterogeneity, the fixed-effect inverse variance-weighted method was applied to pool the effect size; if heterogeneity was observed, a random effects model was employed. A visual inspection of a funnel plot and the use of the trim-and-fill method were used to evaluate publication bias. Statistical analysis was performed using Review Manager version 5.3 (Cochrane Collaboration, Copenhagen, Denmark) and Comprehensive Meta-Analysis version 4 (Biostat, New York, NY, USA).

## 3. Results

The meta-analysis incorporated three studies, totaling 17,421 participants. The number of patients in each selected study ranged from 439 to 16,505 (mean 5807). After excluding patients with clinical stage cT1/a/cisN0M0, 13,009 patients remained for further analysis. The female to male ratio was approximately 1:4. The included studies were performed in three distinct geographical areas: America, Europe, and Asia. All included studies used a cut-off threshold of ≥15 LN as a marker of adequate PLND, with two studies additionally using a threshold of ≥10 LNs. NAC was administered to 15.97% of patients, with the lowest exposure observed in America (14.98%) and the highest in the study from Japan (47.38%). Two studies provided information on the NAC regimens administered. The baseline characteristics of the included studies are summarized in Table 1.

**Figure 2:**
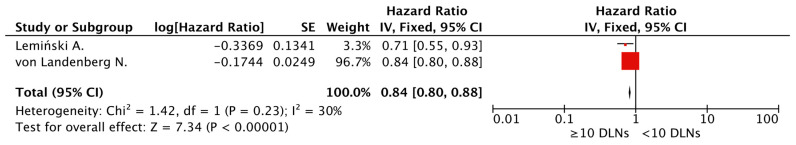
Meta-analysis of overall survival following adequate pelvic lymph dissection (≥10 nodes) [9,11].

**Figure 3:**
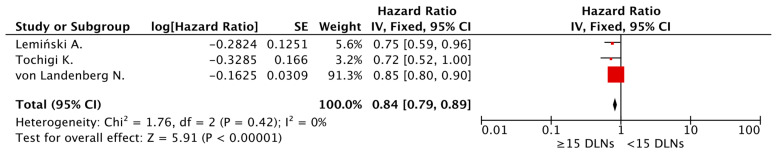
Meta-analysis of overall survival following adequate pelvic lymph dissection (≥15 nodes) [9,10,11].

The analysis of the entire cohort confirmed the prognostic value of both chosen cut-off thresholds for adequate PLND at the time of RC. The pooled HR for the ≥10 LN threshold was 0.84 (95% CI 0.80–0.88), with no observed heterogeneity (*I*^2^ = 30%; Figure 2). The fixed-effect inverse variance-weighted method was applied. The pooled HR for the ≥15 LN threshold was also 0.84 (95% CI 0.79–0.89), with no heterogeneity (*I*^2^ = 0%; Figure 3). The trim-and-fill method suggested that two studies were missing, indicating that the corrected HR was 0.85 (95% CI: 0.81–0.90).

**Figure 4:**
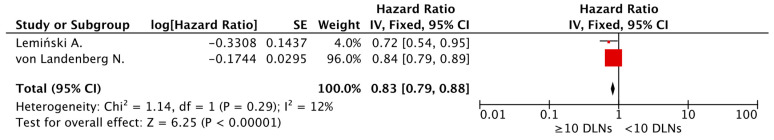
Meta-analysis of overall survival following adequate pelvic lymph dissection (≥10 nodes) in the non-NAC subgroup [9,11].

**Figure 5:**
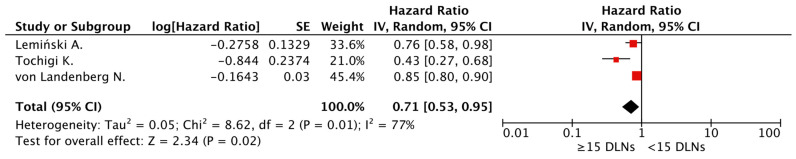
Meta-analysis of overall survival following adequate pelvic lymph dissection (≥15 nodes) in the non-NAC subgroup [9,10,11].

The subgroup analysis of patients with a clinical stage of BC cT2-4aN0M0 who were not exposed to NAC included 10,426 patients. In this subgroup, adequate PLND significantly influenced post-cystectomy survival. The prognostic difference was observed in both evaluated lymph node number cut-off thresholds. Patients with a higher number of resected LN lived longer. The pooled HR for the ≥10 LN threshold was 0.83 (95% CI 0.79–0.88). No heterogeneity was observed (*I*^2^ = 12%; Figure 4). The pooled HR for the ≥15 LN threshold was 0.71 (95% CI 0.53–0.95). The data were heterogeneous (*I*^2^ = 77%; Figure 5), and thus, the random-effects model was applied. Using the trim-and-fill method, the value of HR remained unchanged.

**Figure 6:**
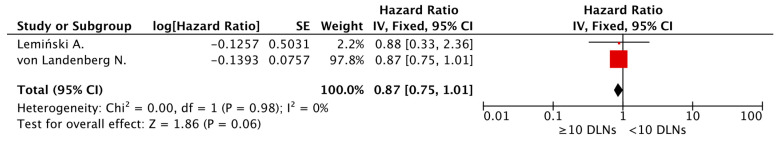
Meta-analysis of overall survival following adequate pelvic lymph dissection (≥10 nodes) in the NAC subgroup [9,11].

**Figure 7:**
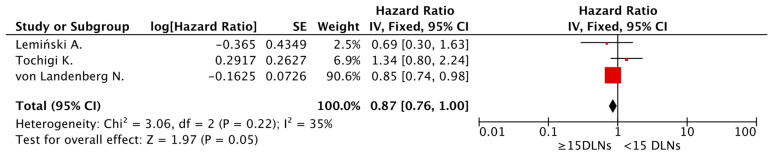
Meta-analysis of overall survival following adequate pelvic lymph dissection (≥15 nodes) in the NAC subgroup [9,10,11].

The subgroup of patients exposed to NAC consisted of 2583 patients. Adequate PLND in patients undergoing RC with preoperative systemic therapy was not associated with a survival benefit. This lack of association was observed for both lymph node number cut-off thresholds. The pooled HR for cut-off thresholds ≥10 and ≥15 LN was 0.87 (95% CI 0.75–1.01; Figure 6) and 0.87 (95% CI 0.76–1.00; Figure 7), respectively. The corresponding *I*^2^ statistic was 0% and 35%, respectively. Using the trim-and-fill method, the HR value remained unchanged.

## 4. Discussion

RC remains the mainstay of treatment for MIBC. The rationale for a lymphadenectomy in MIBC is based on the natural history of the disease. The tumor progressively invades from the bladder mucosa through the muscularis propria to the perivesical fat and adjacent organs. Concurrently, tumor cells may metastasize to regional lymph nodes or distant sites through lymphatics and blood vessels [15]. Consequently, a PLND is currently considered an integral part of RC. However, ongoing debates question how to assess PLND, and which parameter should measure the quality of LN resection. Furthermore, it remains unclear when a patient can be deemed to have undergone an adequate PLND. Numerous authors have tried to address these issues, with some proposing anatomical boundaries as a measurement of quality. Nevertheless, a recent prospective randomized trial of standard versus extended PLND in an RC cohort (LEA AUO AB 25/02) failed to confirm the survival benefit of extended LND [16]. Other authors have suggested that lymph node density might be a valuable independent predictor of clinical outcomes following RC. However, this parameter can only be measured in patients with LN-positive disease. Moreover, there is a significant variability in proposed cut-off thresholds for LN density, ranging from 10 to 25% [17]. Counting the number of LNs dissected during the surgery has also been proposed by several authors, with the most often suggested cut-off thresholds for adequate PLND being the classic approach of 10 LNs and the more extensive approach of 15 LNs [11,18,19]. In our meta-analysis, we included studies that used at least one of these two approaches for adequate PLND. Depending on the definition used, the percentage of patients with adequate PLND in the included studies ranged from 48 to 75%. This indicates that a significant number of patients might not have received proper treatment. Conversely, the receipt of adequate PLND in the included studies aligns with population-based studies. Analysis of data from the Surveillance, Epidemiology, and End Results database revealed that only 45.2% of patients had adequate PLND at the time of RC [19]. Monaghan et al., using data from the National Cancer Database, found 74.4% of adequate PLND in their analysis. However, these authors proposed a more liberal and uncommon assumption of adequate PLND with a cut-off point of only 8 LNs [20]. Despite its prognostic role, even in patients with positive LNs, our observations suggest that adequate PLND is underutilized.

In addition to surgery, the current standard of care for patients with MIBC also includes NAC. A multidisciplinary approach is offered to all patients eligible for cisplatin, meaning that approximately 60% of patients should be exposed to NAC before RC. Preoperative systemic therapy is especially effective in patients with urothelial and neuroendocrine tumors, providing downstaging to <ypT2N0M0 in half of them. Pathological complete remission (pCR) is achievable in one-third of patients [21]. NAC has been shown to significantly improve patient survival, largely attributed to better local control of the tumor. Patients exposed to NAC have a 5–8% higher five-year OS than those who underwent upfront RC [22,23]. However, the survival advantage of NAC may originate not only from local disease control but also from treatment of potential micrometastatic spread of BC. Metastatic disease is diagnosed within two years in nearly half of the patients following RC, significantly reducing patient survival [24]. The median survival of patients with systemic recurrence who undergo salvage cytotoxic chemotherapy is 9–26 months [25,26]. However, a significant proportion of these patients are not suitable candidates for further treatment. Hence, new drugs are increasingly introduced as a salvage approach, with immune checkpoint inhibitors appearing most promising in this setting [27]. The role of NAC in treating micrometastases and preventing distal metastases after RC is analogous to the therapeutic significance of PLND [10]. In our meta-analysis, patients exposed to NAC no longer obtained an oncological benefit from more extensive lymphadenectomy. Comparable results were also observed in other solid tumors, including breast cancer and ovarian cancer, where exposure to NAC led to a significant reduction in the number of axillary lymph node dissections [28,29]. Considering that extended PLND might be associated with adverse outcomes, such as longer hospital stay, increased operating time, greater blood loss, and a higher rate of post-operative complications, limiting the extent of PLND might be a reasonable alternative in patients who received NAC [30]. Restricting the collateral damage of pelvic lymphatic drainage might prevent the development of serious complications, likely without sacrificing oncological outcomes. This might be particularly important in older patients. It has been found that octogenarians who underwent RC with PLND were at a higher risk of specific postoperative complications than those who did not undergo PLND. Significant differences were mainly observed in vascular, wound, pulmonary, and infectious complications, which reflected in the total hospitalization cost, significantly higher for patients aged 80 and above who underwent PLND, compared to those in whom PLND was avoided [31]. Furthermore, Grabbert et al. were unable to show a significant impact of PLND on cancer-specific survival (CSS), OS, or progression-free survival (PFS) in this age group of patients [32]. Comparable results were presented by Larcher et al., who used the SEER-Medicare dataset to identify patients diagnosed with BC and treated with RC alone or RC with PLND. In their study, RC with PLND was linked with improved OS and CSS compared to RC alone in younger, healthier RC candidates, but not in older or sicker patients [33]. Interestingly, contrasting results have been presented by other authors. Abdollah et al. found that the protective effect of PLND on CSS and OS was similar in patients aged 80 and above and those younger than 80 years. Therefore, they concluded that advanced age should not be a limiting factor for PLND at RC. However, this study did not provide information regarding chemotherapy status, and the percentage of patients with a tumor stage of <T2N0M0 was marginal [34]. Given that around 50% of patients show pathological partial remission (<ypT2N0M0) after NAC, a similar result as in Abdollah et al.’s study might not be observed in NAC pre-treated octogenarians.

Despite our meta-analysis showing that PLND does not provide an additional benefit if patients were exposed to NAC before RC, the pooled HRs were on the brink of significance. The *p*-value for cut-off thresholds ≥10 and ≥15 LN was 0.06 and 0.05, respectively, indicating that the NAC cohort also included patients who would benefit from PLND. It seems plausible that not all these patients responded to NAC. However, to confirm this assumption, individual patient data should be available from the included studies, and an analysis of the therapeutic significance of PLND in NAC non-responders should be performed. Additionally, a parallel analysis in patients with pathological partial remission (pPR; ypT1ypNo) and pCR to NAC should be conducted. Results from these analyses would provide insight into in which patients a PLND could safely be constrained or even omitted. Some answers to these questions might be found in previous reports. Nassiri et al. revealed that patients undergoing NAC might still be at risk of occult disease outside of the bladder, despite a clinical complete response diagnosed with computed tomography, cystoscopy, and cytology. They found that occult LN metastasis were present in patients with pCR and pPR in 4.9% and 5.4% of patients, respectively [35]. Furthermore, Kukreja et al. discovered that pT0 status and lack of LN metastasis cannot be reliably recognized by the absence of residual disease (cT0) on transurethral resection of bladder tumor (TURBT) after NAC. Moreover, a significant fraction of patients with cT0 bladders had locally advanced BC and/or LN metastasis in the RC specimen [36]. Additionally, an analysis of the National Cancer Database also revealed that occult regional LN metastasis in patients with pCR depends on the variant histology of BC. Neuroendocrine histology has the lowest risk of persistent LN disease, which could have implications for post-chemiotherapy management [37]. In contrast, Kaag et al. found that no patients who achieved stage ypT0 after NAC had LN involvement at RC or recurrence within the regional LN template. However, in patients who were rendered pT0 at RC/PLND after TURBT alone, LN involvement was observed. The LN-positive rate at RC for these patients was 14%, and 7% of patients developed LN metastasis postoperatively. Kaag et al.’s results indicated high chemoresponsiveness of the regional LNs in patients with ypT0 after NAC and RC [38]. This supports the belief that PLND might be safely omitted in patients with pCR after NAC, assuming that all these patients had an initial clinical tumor stage of cT2N0M0. However, a completely different scenario might be observed in patients with cT3-4N0M0 tumors. These patients have shown superior efficacy of NAC compared to patients with organ-confined disease in terms of OS. However, patients with initial extravesical disease might still have LN involvement after receiving NAC. Mertens et al. found that occult LN micrometastases are present in 20% of these patients despite preoperative chemotherapy. Naturally, the rate of pathological LN involvement is much higher in patients with cT3-4N0M0 BC who undergo upfront RC. Nevertheless, one-fifth of cT3-4N0M0 chemotherapy pre-treated patients would still have persistent nodal disease [39]. Therefore, the approach to abandon PLND in these patients might be unjustified since a significant proportion of patients would not be cured. In our meta-analysis, 22% of patients had cT3-4N0M0 disease. This subgroup of the analyzed population could significantly influence our results. Consequently, the *p*-value for both cut-off thresholds was almost significant in NAC pre-treated patients. This underlines the complexity of this clinical issue and emphasizes the need for future studies to precisely define the patients in whom PLND might be safely abandoned.

The diminished prognostic role of PLND in NAC pre-treated patients might differ depending on the administered chemotherapy regimen. Two studies included in this meta-analysis provided data on the chemotherapy regimens used. In the studies from Poland and Japan, the dominant regimens were ddMVAC and GC, respectively [9,10]. Despite a higher rate of extravesical disease in the Polish study, both reported similar pPR and pCR rates. Additionally, in both studies, adequate PLND did not improve survival in the NAC pre-treated patients. However, the Japanese study only analyzed one out of two thresholds of PLND [10]. To date, no study has assessed the importance of adequate PLND in relation to a specific NAC regimen. Data from the GETUG/AFU V05 VESPER trial indicated that PFS was significantly improved in patients receiving ddMVAC instead of GC in the neoadjuvant setting (HR = 0.70, 95% CI: 0.51–0.96; *p* = 0.025) [40]. These results might suggest that a more substantial reduction in the prognostic role of PLND should be observed in patients previously treated with the ddMVAC regimen. However, the GETUG/AFU V05 VESPER trial should be interpreted with caution. Participants assigned to ddMVAC received more intensive treatment, with six cycles of ddMVAC versus four cycles of GC. This discrepancy might be considered a bias of the trial. Nevertheless, recent studies have shown similar results, with the ddMVAC regimen providing a higher benefit in terms of long-term oncological outcomes compared to other regimens [21]. Additionally, Iyer et al. found no safe cut-off threshold for PLND in patients exposed to GC in a neoadjuvant setting. They revealed that GC pre-treated patients have a survival benefit for each additionally resected LN [41]. This suggests that the adequate PLND cannot be defined when the GC regimen is administered. Recently, a dose-dense (dd) schedule of GC was proposed as an alternative to the standard GC regimen. It was thought that ddGG would shorten the time to surgery and yield better oncological results than standard GC. However, a prospective phase two trial showed that the ddGC regimen had comparable local tumor control to the standard GC regimen [42]. Moreover, a higher-than-expected rate of vascular events led to the early closure of the trial. Considering these factors, we believe that ddMVAC is the regimen of choice if limited PLND is planned during RC.

Our meta-analysis results might be especially useful when introducing minimally invasive techniques for RC and PLND. Lisiński et al. found a significant difference between laparoscopic and open PLND in the number of removed LNs when the open approach was replaced by laparoscopy in RC [43]. Other authors have also shown that the learning curve for laparoscopic PLND is long, and increasing experience improves nodal yield [44]. Moreover, Eden et al. defined that 40 cases of laparoscopic PLND are needed to achieve an acceptable lymphadenectomy-specific complication rate, whereas, in terms of LN yield, they established that the learning curve plateaus after 150 cases, although there is a second, less pronounced increase in the rate of LN yield between cases 390 and 500 [45]. For robotic-assisted RC, it is crucial to treat between 20 and 50 patients to optimize the LN yield [46]. Considering that the number of harvested LNs is lower when introducing minimally invasive techniques in RC, it seems rational to prioritize patients pre-treated with NAC, in which the clinical value of PLND is diminished. This approach allows us to gain experience in PLND without risking oncological outcomes.

This meta-analysis has several limitations. The included data came from non-randomized clinical follow-up studies, resulting in observed heterogeneity in the non-NAC group analysis. Moreover, the studies differed in their approach to addressing the lack of randomization. The study from America, which used data from the National Cancer Database, performed a propensity score analysis [11]. In contrast, Lemiński et al. compared NAC pre-treated patients to a historical cohort when systemic therapy was not routinely offered before RC [9]. Tochigi et al. compared preoperative variables between NAC and non-NAC groups, revealing no discrepancy between the two cohorts [10]. It is noteworthy that one of the included studies was single-center with a relatively short follow-up [9]. Furthermore, none of the included studies provided patient stratifications according to NAC regimens and response to systemic treatment. Consequently, it was not possible to conclude which patient cohort might benefit in particular from a restricted PLND without oncological consequences. Additionally, no study provided information regarding cancer-specific survival, potentially introducing additional bias due to concurrent mortality, significant in this population. Lastly, using lymph node counts as a surrogate marker of sufficient lymphadenectomy may be controversial. The number of removed lymph nodes can vary according to surgical technique and pathological handling [6]. Moreover, there are individual variations in the number of lymph nodes located in the pelvis. Davies et al. found substantial interindividual differences using cadaveric models, with pelvic lymph node counts ranging from 10 to 53 nodes. This indicates the limited utility of lymph node count as a surrogate for dissection extent [47].

## 5. Conclusions

In conclusion, despite these limitations, this meta-analysis indicates a lack of association between adequate PLND and OS in patients undergoing multidisciplinary treatment of MIBC involving NAC and RC. It appears likely that NAC diminishes the therapeutic benefit of adequate PLND. Nonetheless, more extended PLND is needed in patients not exposed to NAC, and in those with locally advanced disease on presentation. Further prospective trial should identify patients who might benefit the most from the limitation of PLND after exposure to NAC.

## Figures and Tables

**Table 1:** Baseline characteristics and quality assessments of included studies.

Study	Country	Recruitment Period (Years)	No. of Patients	Age (Years)	Gender	Clinical Stage	Cut-Off Thresholds of PLND	NAC Regimens	Response to NAC	Follow-Up (months)	Study Design	NOS
von Landenberg N. et al. (2018) [11]	USA	2004–2012	non-NAC: 14031 NAC: 2474	<70: 8585 ≥70: 7920	M: 12549 F: 3956	cT1/a/cis: 4412 cT2: 9635 cT3/T4: 2461	≥10 LN ≥15 LN	n/a	n/a	Md: 55.49 IQR: 34.73–75.96	multi-center study	8
Lemiński A. et al. (2021) [9]	Poland	2003–2020	non-NAC: 356 NAC: 83	non-NAC: Md: 65 (R: 40–88) NAC: Md: 66 (R: 44–80)	M: 342 F: 97	cT2: 170 cT3: 174 cT4: 95	≥10 LN ≥15 LN	ddMAVC: 54 GC: 25 GCa: 4	pCR: 27.71% pPR: 44.57%	Md: 20.73 IQR: 9.467–52.233	single-center study	7
Tochigi K. et al. (2022) [10]	Japan	2004–2019	non-NAC: 251 NAC: 226	non-NAC: Md: 71 (R: 36–89) NAC: Md: 70 (R: 44–85)	M: 336 F: 111	cT2: 287 cT3: 154 cT4: 36	≥15 LN	MVAC: 6 ddMAVC: 17 GC: 185 GCa: 14 GC + GCa: 4	pCR: 21.68% pPR: 46.46%	Md: 41 R: 1–194	multi-center study	8

ddMVAC: dose-dense methotrexate, vinblastine, doxorubicin, and cisplatin; F: female; GC: gemcitabine and cisplatin; GCa: gemcitabine and carboplatin; IQR: interquartile range; LN: lymph nodes; Md: median; M: male; MVAC: methotrexate, vinblastine, doxorubicin, and cisplatin; NAC: neoadjuvant chemotherapy; n/a: not available; NOS: Newcastle-Ottawa Scale; pCR: pathological complete response; pPR: pathological partial response; PLND: pelvic lymph node dissection; R: range.

## Data Availability

Any data reported have been appropriately referenced, with no original data being reported by the authors.
